# The Relationship of Mitral Annular Calcification and Aortic Valve Sclerosis to Endothelial Dysfunction

**DOI:** 10.7759/cureus.59712

**Published:** 2024-05-06

**Authors:** Mohammed N Elganainy, Mohamad H Farrag, Ehab N Selim, Amr A Sherif, Amr F Elhadidy, Ahmed A Elheet

**Affiliations:** 1 Cardiovascular Medicine, National Heart Institute, Cairo, EGY; 2 Cardiology, Alhada Armed Forces Hospital, Taif, SAU; 3 Cardiovascular Disease, National Heart Institute, Cairo, EGY; 4 Cardiovascular Disease, Alhada and Taif Armed Forces Hospitals, Taif, SAU

**Keywords:** vascular ultrasound, echocardiography, carotid intima-media thickness, endothelial dysfunction, aortic valve sclerosis, mitral annular calcification

## Abstract

Background: Calcific aortic valve disease (CAVD) and mitral annular calcification (MAC) are associated with various cardiovascular diseases and may influence systemic vascular pathologies. However, their relationship with endothelial dysfunction and carotid intima-media thickness (CIMT) remains poorly elucidated. This research aims to explore the associations between MAC, aortic valve sclerosis (AVS), and markers of vascular dysfunction, specifically CIMT and endothelial function.

Methods: This prospective observational study included 200 patients undergoing routine echocardiographic evaluation at the National Heart Institute between May 2022 and April 2023. Patients were stratified into four groups namely isolated MAC (38 patients), isolated AVS (72 patients), combined MAC and AVS (50 patients), and a control group without MAC or AVS (40 patients). All participants underwent comprehensive cardiovascular evaluation, including transthoracic echocardiography (TTE) and carotid duplex ultrasonography. Endothelial function was determined by measuring reactive hyperemia-induced alterations in brachial artery diameter.

Results: The mean age of participants was 60.6±8.4 years, with a predominance of male subjects (64%). No significant differences were noted in baseline demographic and clinical characteristics across the groups. Patients with isolated AVS, isolated MAC, and both conditions demonstrated increased CIMT compared to controls, with significant differences noted in the combined MAC and AVS group compared to controls (p-value=0.031). Endothelial dysfunction was observed in 14.8% of the AVS group and 21.1% in the combined group, but no significant differences existed when compared to controls. The study also revealed that patients with AVS are more likely to exhibit increased CIMT (p-value=0.008).

Conclusions: Both MAC and AVS are connected to increased CIMT, suggesting a link with systemic atherosclerotic processes. Although the existence of endothelial dysfunction was not significantly higher in patients with valvular calcifications, the findings support the need for further research into the cardiovascular implications of CAVD and MAC.

## Introduction

Atherosclerosis is a systemic inflammatory disease that preferentially affects specific anatomic sites [1]. An early sign of atherosclerosis is a malfunction of the systemic endothelium. Many arteries' endothelium, a monolayer of cells with a variety of vital roles, is first affected by atherosclerosis. Numerous medical diseases can cause endothelial dysfunction, the most common of which is atherosclerosis. Additionally, patients with diabetes, heart failure, hypertension, high cholesterol, cigarette smoking, and aging are observed to have it [2].

Improving endothelial function is a desirable therapeutic goal, and several interventions have shown promise in this area. However, only a few of these interventions, such as statins, have been systematically evaluated in large, prospective, randomized controlled studies [3]. Aortic valve calcification (AVC) and mitral annular calcification (MAC) are more common in older adults and more common in populations with severe atherosclerosis. According to earlier pathological research, they are a sign of a degenerative process that gets worse with age. Aortic atheroma, peripheral arterial atherosclerotic disease, and coronary artery disease have all been shown to be significantly correlated with MAC in a number of ultrasound cardiovascular studies, supporting this theory. Conduction abnormalities and atrial fibrillation are also linked to MAC [3].

Systolic ejection murmurs, which can be found incidentally on echocardiography or as a physical examination finding, are the two most common ways that aortic sclerosis is identified. The disorder is asymptomatic. In middle-aged adults, the prevalence of aortic sclerosis is less than 10%; in adults 65 years of age and above, it exceeds 25%. Clinical risk factors for atherosclerosis such as diabetes, hypertension, smoking, and hyperlipidemia have been linked to aortic valve sclerosis (AVS). It is a marker for increased cardiovascular risk and can proceed to aortic stenosis; thus, a complete history and physical examination are necessary to identify cardiovascular risk factors. Flow-mediated dilation (FMD) is the mechanism by which blood vessels widen in response to shear stress; quantifying FMD is a clinical technique for assessing endothelial function. Early on in atherosclerotic vascular disease, FMD impairment is seen. Brachial artery occlusion causes the endothelium to produce nitric oxide, which causes vasodilation that may be measured and visualized as a measure of vasomotor function [2].

When reactive hyperemia is present, ultrasound imaging of the brachial artery is a commonly utilized method to measure endothelium-dependent vasomotion and determine whether endothelial dysfunction is present. A widespread disease process known as impaired endothelium-dependent vasomotion causes aberrant blood vessel tone regulation and the loss of several atheroprotective properties of healthy endothelium [4,5]. A further indicator of future cardiovascular risk is impaired peripheral endothelial function, particularly when excluding coronary heart disease [2].

There is evidence to suggest that endothelial dysfunction detected on coronary angiography following cholinergic injection may be correlated with endothelial dysfunction shown on brachial artery ultrasound imaging [6]. This association may have an unclear cause. Studies of atherosclerosis frequently include carotid intima-media thickness (CIMT) measurements. In response to variations in shear and tensile stress, some researchers have proposed that an elevated IMT represents a non-atherosclerotic adaptive response [7]. One of the exclusion criteria is end-stage renal disease (ESRD) since patients with ESRD frequently have calcifications in their heart tissues [8].

Our study aims to investigate the correlation between the degree of endothelial dysfunction if any, and calcification at the annuli of the mitral and/or aortic valves. Additionally, we want to look into how MAC, AVC, and CIMT are related to one another.

## Materials and methods

Study population

This research comprised 200 patients recruited from the patients undergoing echocardiographic evaluation at the National Heart Institute, Cairo, Egypt, in the period between May 2022 and April 2023.

The study protocol was reviewed and approved by The Ethics Committee of the Cardiovascular Department, National Heart Institute, Egypt (Approval number: NHI230723). Informed written consent was obtained from all participants included.

In the study, participants were categorized based on their echocardiographic findings into four distinct groups as follows (Table 1).

**Table 1:** Classification of study participants based on echocardiographic findings TTE: transthoracic echocardiography; MAC: mitral annular calcification; AVS: aortic valve sclerosis

Group	Description	Number of patients	Diagnostic criteria
I - Isolated mitral annular calcification (MAC)	Patients diagnosed with isolated MAC	38	TTE showing an intense echo-producing structure at the intersection of the posterior mitral valve leaflet and the atrioventricular groove
II - Isolated aortic valve sclerosis (AVS)	Patients with isolated AVS	72	TTE showing transaortic flow velocity < 2.5 m/s, enhanced echogenicity, and thickening of aortic valve leaflets without restricting leaflet motion
III - Combined MAC and AVS	Patients exhibiting both MAC and AVS	50	Criteria from both groups I and II
IV - Control group	Patients with neither MAC nor AVS	40	Absence of echocardiographic findings characteristic of groups I, II, or III

Exclusion criteria

Patients were excluded if they had a poor echocardiographic window, valvular heart disease other than MAC or AVS, or end-stage renal disease (ESRD).

Methods

All patients were exposed to 1) Comprehensive history and physical assessment (a history of peripheral vascular disease and coronary artery disease, obesity, smoking, dyslipidemia, diabetes mellitus, and smoking should be taken into consideration). 2) An electrocardiogram was used to detect any indications of CAD, rhythm abnormalities, axis deviation, or chamber enlargement, and a 12-lead ECG was performed. 3) A complete echocardiographic evaluation was performed on all patients via transthoracic echocardiography (TTE), which included the utilization of 2D, M-mode, and color flow mapping to detect aortomitral calcification and sclerosis. Scans of the parasternal short/long axis and apical four to five chambers.

The TTE criteria for MAC were the presence of a strong echo-producing structure on the parasternal long-axis, apical four-chamber, or parasternal short-axis view, situated at the intersection of the atrioventricular groove and posterior mitral valve leaflet [2]. With a transaortic flow velocity of fewer than 2.5 m/s on TTE, AVS was characterized by thickening and increased echogenicity of the aortic valve leaflets, but no restriction of leaflet motion [2].

Standard parasternal and apical views were incorporated into each examination in accordance with the guidelines set forth by the American Society of Echocardiography (ASE). Every measurement was acquired using three cycles, with the exclusion of post-ectopic beats.

The following were calculated namely ejection fraction, diastolic function, left ventricular (LV) end-systolic and end-diastolic dimensions, and wall motion asynergy (WMA).

4) Carotid and peripheral arterial duplex were done using an ultrasound machine using the 7.5 MHz linear transducer to evaluate the endothelial function study and intima-media thickness of the common carotid artery.

For the endothelial function study, patients were told to lie in a supine position in silence for 10 minutes before the study. The measurement of the brachial artery's diameter was obtained by analyzing two-dimensional ultrasound pictures. Scans were performed both at rest and during reactive hyperemia in each investigation. A longitudinal segment of the brachial artery was performed on the dominant arm, positioned 2 cm to 15 cm anterior to the elbow. To optimize images of the lumen-arterial wall interface, the focus zone was adjusted, and machine-operating parameters remained constant throughout the remainder of the investigation.

A constant distance was maintained between the artery diameter and an anatomical marker, such as a bifurcation. After obtaining the initial readings, a pneumatic tourniquet was inflated to a pressure of 250 mm Hg below the elbow. The forearm cuff was occluded for a duration of 4.5 minutes. After 4.5 minutes of cuff occlusion, sustained maximal vascular dilation and maximal flow change often occur; a longer cuff occlusion time does not result in a stronger response. A reduced time period of cuff occlusion results in a diminished stimulus intensity and fails to induce sustained vasodilation one minute following the release of the cuff. Hence, the artery diameter was assessed one minute subsequent to the deflation of the cuff.

The intima-media thickness of the common carotid artery was assessed 1 cm proximal to the carotid bifurcation by directing the ultrasound beam perpendicular to the posterior wall of the vessel to obtain two parallel echogenic lines representing the blood-intima and the intima-media interfaces; then, the IMT was assessed as the distance between the two lines, three readings were obtained, and the average was taken [9,10].

Statistical analysis

For testing data normality Shapiro test was used. Data were statistically described in terms of mean ± standard deviation (SD), or frequencies (number of cases) and percentages when appropriate. Comparison of numerical variables between the study groups was performed using Student's t-test for comparing two groups and one-way analysis of variance (ANOVA) for more than two groups. For categorical data, the chi-square (χ²) test was used. When the expected frequency was less than five, the Fisher exact test was employed. P-values less than 0.05 were considered statistically significant. All statistical calculations were performed using the SPSS for Windows, Version 15 (Released 2006; SPSS Inc., Chicago, United States) for Microsoft Windows.

## Results

This study is a comparative cross-sectional investigation that enrolled 200 consecutive patients undergoing echocardiographic evaluations at the National Heart Institute, Cairo, Egypt, from May 2022 to April 2023. We explored the relationship between MAC and/or AVS with endothelial dysfunction, frequent risk factors for CAD and atherosclerosis, as well as their association with a rise in CIMT.

Patients were split into four groups. Group I: 38 patients with isolated MAC, group II: 72 patients with isolated AVS, group III: 50 patients with both MAC and AVS, and group IV: 40 patients without MAC or AVS (control group).

Clinical characteristics of the study population

We compared these four groups based on demographic data and found significant differences as shown in Table 2.

**Table 2:** Clinical characteristics of the study population Data were presented as mean±SD and N (%). SD: standard deviation; DM: diabetes mellitus; HTN: hypertension; H/O: history of; CAD: coronary artery disease

Variable	Group I	Group II	Group III	Group IV
Age (mean±SD)	62.21±8.12	61.07±8.32	64.58±9.32	56.67±4.14
Male	32 (64.3%)	50 (70.4%)	29 (57.9%)	21 (53.3%)
Female	13.3 (35.7%)	21 (29.6%)	21 (42.1%)	18 (46.7%)
DM (yes)	24.4 (64.3%)	21 (29.6%)	26 (52.6%)	21 (53.3%)
HTN (yes)	32 (64.3%)	45 (63%)	37 (73.7%)	21 (53.3%)
H/O Smoking (yes)	16 (42.9%)	32 (44.4%)	16 (31.6%)	21 (53.3%)
Dyslipidemia (yes)	32 (85.7%)	59 (81.5%)	47 (94.7%)	21 (53.3%)
H/O CAD (yes)	21 (57.1%)	59 (81.5%)	34 (68.4%)	21 (53.3%)

There was no significant variation between the four groups concerning gender, age, smoking, hypertension, the existence of dyslipidemia, diabetes, and history of CAD. However, patients in group III were more prone to possessing dyslipidemia contrasted with those in group IV (p-value=0.011, Table 3).

**Table 3:** Comparison between MAC and AVS Data were presented as mean±SD and N (%). SD: standard deviation; MAC: mitral annular calcification; AVS: aortic valve sclerosis, ECG: electrocardiogram; H/O: history of; CAD: coronary artery disease; LVEDD: left ventricular end-diastolic diameter; LVESD: left ventricular end-systolic diameter; FS: fractional shortening; EF: ejection fraction; DF: diastolic function; RWMA: regional wall motion abnormalities; CCA-IMT: common carotid artery intima-media thickness

Variable	Group I (MAC)	Group II (AVS)	p-value
Age (years)	62.21±8.116	61.07±8.32	0.677
Males	32 (64.3%)	50 (70.4%)	0.73
Smoking	16 (42.9%)	32 (44.4%)	<0.999
Hypertension	32 (64.3%)	45 (63%)	<0.999
Dyslipidemia	32 (85.7%)	59 (81.5%)	<0.999
Diabetes	24.4 (64.3%)	21 (29.6%)	0.048
H/O CAD	21 (57.1%)	59 (81.5%)	0.14
ECG			
Axis (normal)	13 (35.7%)	24 (33.3%)	<0.999
ST-T wave changes (yes)	24 (64.3%)	21(55.6%)	0.742
ECHO			
LVEDD (cm)	5.15±1.09	5.47±0.78	0.28
LVESD (cm)	3.6±1.16	3.82±1.02	0.54
FS	30.5±8.13	30.19±8.78	0.91
EF	57.43±11.55	55.67±14.09	0.69
Diastolic Function			
Normal DF	11 (28.6%)	13 (18.5%)	0.69
Impaired DF	27 (71.4%)	59 (81.5%)	
RWMA	13 (35.7%)	35 (48.1%)	0.52
CCA-IMT (mm)	0.89±0.17	0.99±0.28	0.28
Endothelial dysfunction	5 (14.3%)	11 (14.8%)	<0.999

Clinical characteristics

MAC and AVS were present predominantly in patients around the age of 60, more commonly in males than in females, and were more prevalent among patients with risk factors for atherosclerosis such as hypertension, diabetes, and dyslipidemia.

MAC patients exhibited a higher propensity to be diabetic compared to patients with AVS (p-value=0.048, Table 3).

ECG findings

Axis and ST-T wave variations were not significantly different between the two groups in terms of (ECG) findings (Table 3).

In group I, 32 patients (85.7%) had normal LV diameters. In group II, 53 patients (74.1%) exhibited normal LV diameters. Normal LV contractility was observed in 27 patients (71.4%) in group I and 42 patients (59.3%) in group II. Diastolic dysfunction was present in 27 patients (71.4%) in group I and 59 patients (81.5%) in group II.

However, no significant variations were observed between the groups regarding echocardiographic findings (LV end-diastolic diameter (EDD), LV end-systolic diameter (ESD), LV systolic and diastolic functions, and regional wall motion abnormalities (RWMAs)). Notably, a high percentage of patients in groups I, II, and III showed diastolic dysfunction in their echocardiographic examinations (Table 3).

Common carotid intima-media thickness

In group I, 27 patients (71.4%) had increased common carotid artery IMT. In group II, 51 patients (74.1%) showed increased thickness. In contrast, there was no statistically significant distinction observed between the two groups in terms of elevated common CIMT (Table 3).

Endothelial function study

In group I, 5 patients (14.3%) had endothelial dysfunction, and 33 patients (85.7%) had normal endothelial function. In group II, 10 patients (14.8%) exhibited endothelial dysfunction, and 62 patients (85.2%) had normal function. No substantial distinction was observed between the two groups with respect to endothelial dysfunction (Table 3).

In relation to endothelial dysfunction and increased CCA-IMT, no statistically significant distinction was observed between the two groups (Table 4).

**Table 4:** Comparison between MAC and control Data were presented as mean±SD and N (%). MAC: mitral annular calcification; CCA-IMT: common carotid artery intima-media thickness; SD: standard deviation

Variable	Group I (MAC)		Group IV (control)	p-value
Increased CCA-IMT	27 (71.4%)		24 (60%)	0.7
CCA-IMT (mm)		0.89±0.16	0.85±0.13	0.35
Endothelial dysfunction	5 (14.3%)		0 (0%)	0.224

No significant disparity was seen between the two groups with respect to increased CCA-IMT and endothelial dysfunction (Table 5).

**Table 5:** Comparison between AVS and control Data were presented as mean±SD and N (%). AVS: aortic valve sclerosis, CCA-IMT: common carotid artery intima-media thickness; SD: standard deviation

Variable	Group II (AVS)	Group IV (control)	p-value
Increased CCA-IMT	53 (74.1%)	9 (60%)	0.49
	0.99±0.28	0.85±0.13	0.71
Endothelial dysfunction	11 (14.8%)	0 (0%)	0.28

No significant disparity was seen between the two groups with respect to increased CCA-IMT and endothelial dysfunction (Table 6).

**Table 6:** Comparison between both MAC-AVS and control Data were presented as mean±SD and N (%). MAC: mitral annular calcification; AVS: aortic valve sclerosis; CCA-IMT: common carotid artery intima-media thickness; SD: standard deviation

Variable	Group III (both)	Group IV (control)	p-value
Increased CCA-IMT	24 (47.4%)	9 (60%)	0.51
	0.88±0.24	0.85±0.13	0.66
Endothelial dysfunction	10 (21.1%)	0 (0%)	0.113

Increased common carotid intima-media thickness

Patients with AVS demonstrated a statistically significant correlation with increased IMT in the common carotid artery compared to patients without AVS (p-value=0.008). Similarly, patients with both MAC and AVS showed a statistically significant correlation with increased IMT in the common carotid artery in contrast to patients in the control group and those with isolated MAC or isolated AVS (p-value=0.031).

## Discussion

The primary objectives of this research endeavor were to assess the clinical and echocardiographic attributes of individuals diagnosed with MAC and/or AVS, as well as to investigate the correlation between endothelial dysfunction severity, if present, and calcification at the mitral and/or aortic valve annuli. Furthermore, we aimed to investigate the correlation between MAC and AVS in connection to CIMT and prevalent risk factors associated with atherosclerosis and CAD.

We examined the correlation between MAC and/or AVS and the prevalent atherosclerotic risk factors among our patients. Our research revealed that MAC and AVS were most frequently observed in senior individuals, namely in males (61±8 SD), who had risk factors for atherosclerosis including hypertension, diabetes, and dyslipidemia. Furthermore, these conditions were more prevalent in males than in females.

These conclusions are consistent with those of several studies investigated in the same field. To illustrate, Boon et al. [11] examined the prevalence with which patients with mitral annular and AVCs have atherosclerotic risk factors. They concluded that these calcifications should be regarded as manifestations of generalized atherosclerosis due to their association with atherosclerotic risk factors.

Similarly, Allison et al. [1] employed electron-beam computed tomography to assess 1,242 consecutive asymptomatic patients free of clinical coronary heart disease to quantify the amount of calcium deposition brought on by atherosclerosis in five different vascular beds, including the mitral and aortic annuli. The two conventional cardiovascular risk factors that were found to be independently linked to the prevalence of calcification in these areas were age and a history of hypertension. Agmon et al. [12] evaluated 381 patients using transthoracic and transesophageal echocardiography to look for signs of aortic regurgitation (AR), high transaortic flow velocities, and AVS. They came to the conclusion that aortic atherosclerosis and various atherosclerosis risk factors are independently linked to AVS in the general population, hence bolstering the theory that AVS is an aortic valve-related disease similar to atherosclerosis.

Diabetes was more common in patients with MAC (p-value=0.048), and prior clinical research has shown a strong correlation between MAC and atherosclerotic risk factors such as hypertension, diabetes, and hyperlipidemia [13,14]. Regrettably, comparable studies on the pathogenesis of calcification at the mitral annuli are rare because of their anatomical placements [1].

Regarding ECG findings in the current study, we discovered a statistically significant connection between aberrant axis deviation and abnormal ST-T alterations in the ECG and calcification at the mitral and/or aortic annulus. As far as we know, this problem has not been explicitly studied in any prior research.

In terms of echocardiographic findings, we observed that patients with calcification at the mitral and/or aortic annulus did not demonstrate statistically significant differences in echocardiographic parameters, including LV diameters, systolic and diastolic functions, and regional wall motion abnormalities (RWMAs). However, during their echocardiographic assessments, a significant proportion of these patients showed signs of severe diastolic dysfunction.

As regards AVS and some echocardiographic parameters, our results contrast with those of Hussain et al. [15], who reported that adults with AVS presented with echocardiographic indicators of preclinical cardiovascular disease, such as incorrect LV geometry, increased left atrial size, aortic valve regurgitation, mitral annular ring calcification, aortic root calcification, and higher pressure gradients and blood flow velocity across the aortic valve, all of which may worsen the prognosis linked to AVS. The discrepancy in our findings could be attributed to the restricted quantity of patients in each group in our study.

As regards MAC and some echocardiographic parameters, approximately 73% of patients with MAC in our study showed diastolic dysfunction, aligning with the findings of Movahed et al. [16], who discovered an independent correlation between MAC and substantial structural cardiac anomalies. This implies that the detection of MAC may function as a marker for other structural heart problems. Our results corroborate their findings regarding the presence of diastolic dysfunction.

With respect to increased common CIMT, our findings indicate a statistically significant connection between AVS and increased IMT in the common carotid artery compared to patients without AVS (p-value=0.008). Additionally, patients with both MAC and AVS exhibited a statistically significant increase in IMT in contrast to patients in the control group and those with isolated MAC or AVS (p-value=0.031).

These results are consistent with those of Sgorbini et al. [3] who assessed 102 patients undergoing TTE and carotid artery echo Doppler for various reasons and found the highest patient scores for valvular/annular calcification and mean cIMT. This suggests that aortic or mitral valve calcification should be seen as indicators of widespread atherosclerosis rather than just normal aging correlations.

Similarly, Yamaura et al. [17] investigated the link between early subclinical AVS and IMT in 252 healthy people using echocardiography and carotid ultrasonography. They found that subjects with AVS had considerably higher carotid IMT than those without. 

Additionally, our findings align with those of Allison et al. [1] who, following correction for cardiovascular disease risk variables, showed that participants with aortic annular calcium present in the thoracic aorta had the highest odds (p-value=0.01), whereas those with MAC in the abdominal aorta had the highest odds (p-value=0.01). Once the presence of calcium in other vascular beds was taken into account, there was a significant correlation found between the increases in calcium in the aortic annulus and the iliac arteries (p-value=0.01) and abdominal aorta (p-value=0.01). Conversely, there was a substantial (p-value=0.02) correlation between calcium in the mitral annulus and the thoracic aorta. These findings imply that atherosclerosis and annular calcification are related, and calcium levels in either annulus could be a helpful screening marker for atherosclerosis.

With respect to endothelial function, in our study, patients with aortic annulus calcification and those with mitral annulus calcification did not vary statistically in the presence of endothelial dysfunction. As far as we are aware, no earlier research has explicitly addressed this problem. We found no statistically significant difference in endothelial dysfunction between patients with AVS and the control group in relation to the association between AVS and endothelial dysfunction. This discovery is in opposition to Poggianti et al. findings [2] who identified a significant association between AVS and systemic endothelial dysfunction in a study involving 102 hospitalized patients (76 men and 26 women; mean age 63.5±9.7 years) with suspected or known coronary artery disease (CAD). Following a referral to a stress echocardiography facility, these patients were scheduled for coronary angiography, revealing a significant association of AVS with systemic endothelial dysfunction (p-value=0.01). Our differing results may be attributed to the fact that 86% of our participants, who were admitted to the cardiovascular department, were undergoing anti-ischemic therapy, which has been shown to impact endothelial function, at the time of testing [2]. However, withholding therapy from all patients would have been impractical.

Finally, this study had some limitations as it was a single-center study with a relatively small sample size, which might limit the generalizability of our findings. Hence, future multicenter studies with larger sample sizes are needed to validate our results.

## Conclusions

This study establishes a significant association between MAC, AVS, and increased CIMT, suggesting their utility in the early detection of systemic atherosclerotic processes. While the presence of endothelial dysfunction was not significantly higher in patients with valvular calcifications compared to controls, our findings underscore the importance of considering MAC and AVS in cardiovascular risk assessments. Given the links between valvular calcifications and increased CIMT, we recommend that clinicians consider incorporating the evaluation of MAC and AVS into routine cardiovascular risk assessments, particularly for patients presenting with other atherosclerotic risk factors. This approach may enhance early identification of individuals at elevated risk of cardiovascular events.

Further research is needed to explore the mechanisms underlying the association between valvular calcifications and systemic atherosclerosis. Prospective studies focusing on the progression of MAC and AVS and their impact on cardiovascular outcomes would be valuable. Additionally, intervention studies could assess the efficacy of targeted therapies in patients with MAC and AVS to mitigate the progression of systemic atherosclerosis.
